# Regulation of transport processes across the tonoplast

**DOI:** 10.3389/fpls.2014.00460

**Published:** 2014-09-10

**Authors:** H. Ekkehard Neuhaus, Oliver Trentmann

**Affiliations:** Plant Physiology, Department of Biology, Technische Universität KaiserslauternKaiserslautern, Germany

**Keywords:** tonoplast, protein phosphorylation, vacuole, tonoplast monosachharide transporters, regulation

## Abstract

In plants, the vacuole builds up the cellular turgor and represents an important component in cellular responses to diverse stress stimuli. Rapid volume changes of cells, particularly of motor cells, like guard cells, are caused by variation of osmolytes and consequently of the water contents in the vacuole. Moreover, directed solute uptake into or release out of the large central vacuole allows adaptation of cytosolic metabolite levels according to the current physiological requirements and specific cellular demands. Therefore, solute passage across the vacuolar membrane, the tonoplast, has to be tightly regulated. Important principles in vacuolar transport regulation are changes of tonoplast transport protein abundances by differential expression of genes or changes of their activities, e.g., due to post-translational modification or by interacting proteins. Because vacuolar transport is in most cases driven by an electro-chemical gradient altered activities of tonoplast proton pumps significantly influence vacuolar transport capacities. Intense studies on individual tonoplast proteins but also unbiased system biological approaches have provided important insights into the regulation of vacuolar transport. This short review refers to selected examples of tonoplast proteins and their regulation, with special focus on protein phosphorylation.

## INTRODUCTION

The vacuole represents a characteristic organelle of plant cells and formerly two types of vacuoles were distinguished: protein storage vacuoles and lytic vacuoles. However, this classification is rather artificial and several facts and recent data suggest that it is not as clear as previously assumed. E.g., in one cell, storage, and lytic vacuoles can exist in parallel and also their total number is quite variable and ranges from many small organelles to only one large central vacuole that can occupy up to 80% of the cell volume. Moreover, differentiation, and hence classification of vacuoles (e.g., by marker proteins or interior solutes) becomes further complicated by the fact that vacuolar protein compositions and stored contents show broad variation not only during development or in different tissues but also in short-term response to different environmental conditions, stressors etc. Due to the broad spectrum of variations vacuoles can be considered as multifunctional organelles involved in diverse cellular processes and adapted to all stages of a plants life.

Vacuoles represent cellular storages for primary metabolites, proteins, pigments, metal, and nonmetal ions, they allow sequestration of xenobiotics and other toxic compounds, and generally are an essential component for the constitution of the cellular turgor pressure. The tonoplast harbors various membrane proteins: proton pumps, channel proteins, ABC transporters, tonoplast intrinsic proteins (TIPs) and diverse solute carriers. The relative abundance of these proteins and their respective activities/regulation determine the specific function of plant vacuoles either in adjustment of cellular – mainly cytosolic – metabolite levels (homeostasis), in detoxification, in plant growth, and plant movement (e.g., of motor cells) or in plant adaptation to abiotic stress situations (e.g., cold and drought). During the past decades, the biochemical properties and physiological functions of several tonoplast proteins have been elucidated (Martinoia et al., 2007, 2012; Neuhaus, 2007). Furthermore, proteome studies provided detailed insights into the complex protein composition of the tonoplast, including tonoplast protein phosphorylation (Carter et al., 2004; Shimaoka et al., 2004; Szponarski et al., 2004; Endler et al., 2006, 2009; Jaquinod et al., 2007; Schmidt et al., 2007; Whiteman et al., 2008a,b) and first studies were performed to discover changes in tonoplast protein compositions caused by environmental changes (Barkla et al., 2009; Schulze et al., 2012).

This review gives special attention to the latest knowledge about the regulation of tonoplast protein activities, particularly by phosphorylation, because these factors represent important control mechanisms in fast adaptation of specific functional properties.

## PHOSPHORYLATION OF TONOPLAST PROTEINS

To maintain vacuolar flexibility transport processes across the tonoplast have to be constantly controlled and altered. This can be achieved by modified expression of genes encoding tonoplast proteins. Corresponding studies have been carried out for example with genes encoding V-ATPase subunits (Dietz et al., 2001; Kluge et al., 2003; Hanitzsch et al., 2007). However, recent research provided evidence that also post-translational modifications of tonoplast proteins represent a fundamental principle in vacuolar transport regulation and adaptation. It seems quite obvious that direct post-translational modifications of the protein allow faster adaptation of protein activity when compared to more initial changes at the pre-protein level (e.g., altered gene expression, transcriptional regulation). Reversible protein phosphorylation by specific protein kinases/phosphatases is a very common post-translational modification and apparently is also relevant for vacuolar transport regulation. Modern mass spectrometry in combination with immobilized metal ion- or titanium dioxide based affinity chromatography offers a feasible method for identification of phosphorylated proteins in enriched tonoplast fractions. Accordingly phosphoproteome studies of tonoplast preparations from various plants led to the identification of several phosphorylated tonoplast proteins (Whiteman et al., 2008a,b; Endler et al., 2009), including proton pumps, aquaporins, Na^+^/H^+^ antiporters and sugar transporters.

The first regulatory phosphorylation of a tonoplast protein was discovered nearly 20 years ago. Maurel et al. (1995) demonstrated that the seed specific aquaporin α-TIP (tonoplast intrinsic protein) forms a water channel and that its water transport activity is regulated by phosphorylation of three serine residues. Corresponding transport activity assays were carried out after expression of wild type or mutated genes (triplets encoding the serine residues) in *Xenopus oocytes* and phosphorylation of the heterologously expressed protein was demonstrated with the help of bovine protein kinase A (PKA; Maurel et al., 1995).

## *Arabidopsis* TONOPLAST SUGAR TRANSPORTERS (TMTs) ARE REGULATED BY A MITOGEN-ACTIVATED TRIPLE KINASE (MAPKKK)

Phosphoproteome studies paved the way to investigate the impact of phosphorylation on tonoplast protein activity/function, like it has been performed with tonoplast monosaccharide transporters (TMT) from *Arabidopsis*. In phylogenetic studies of plant sugar and sugar alcohol transporters, TMTs form a distinct subgroup that comprises three members all targeted to the tonoplast membrane, the TMTs (Büttner and Sauer, 2000, Wormit et al., 2006). A characteristic feature of TMTs is the presence of an extraordinarily large hydrophilic loop between transmembrane domain six and seven. With approximately 320 amino acid residues in length it is about three to four times larger than the corresponding hydrophilic domain of all remaining proteins of the sugar transporter family (Wormit et al., 2006). For a long time it was unclear why plant TMTs established such an extended loop region. However, *in silico* studies led to the identification of several putative phosphorylation sites in the loop region providing first hints for post-translational protein modification in this extended domain. During the past 6 years diverse analyses verified that phosphorylation of the TMT loop actually occurs *in vivo.* The barley ortholog(s) *Hv*STP1/2 of the *Arabidopsis* TMTs exhibit(s) 12 phosphorylated loop residues (Endler et al., 2009). Five of these 12 phosphosites are conserved among the TMT from *Arabidopsis* and barley. However, several are species specific. So far, at least two of the *Arabidopsis*-specific phosphosites were proven to become phosphorylated (Whiteman et al., 2008b; Schulze et al., 2012). After phosphorylation of the TMT-loop was clarified, the following questions immediately came to mind: which kinases catalyze phosphorylation of the TMT-loop? Does protein phosphorylation alter transport properties/activities of the TMT? What are the physiological consequences of TMT phosphorylation? Which conditions (metabolic, environmental etc.) cause alterations in the phosphorylation state of the loop?

**FIGURE 1:**
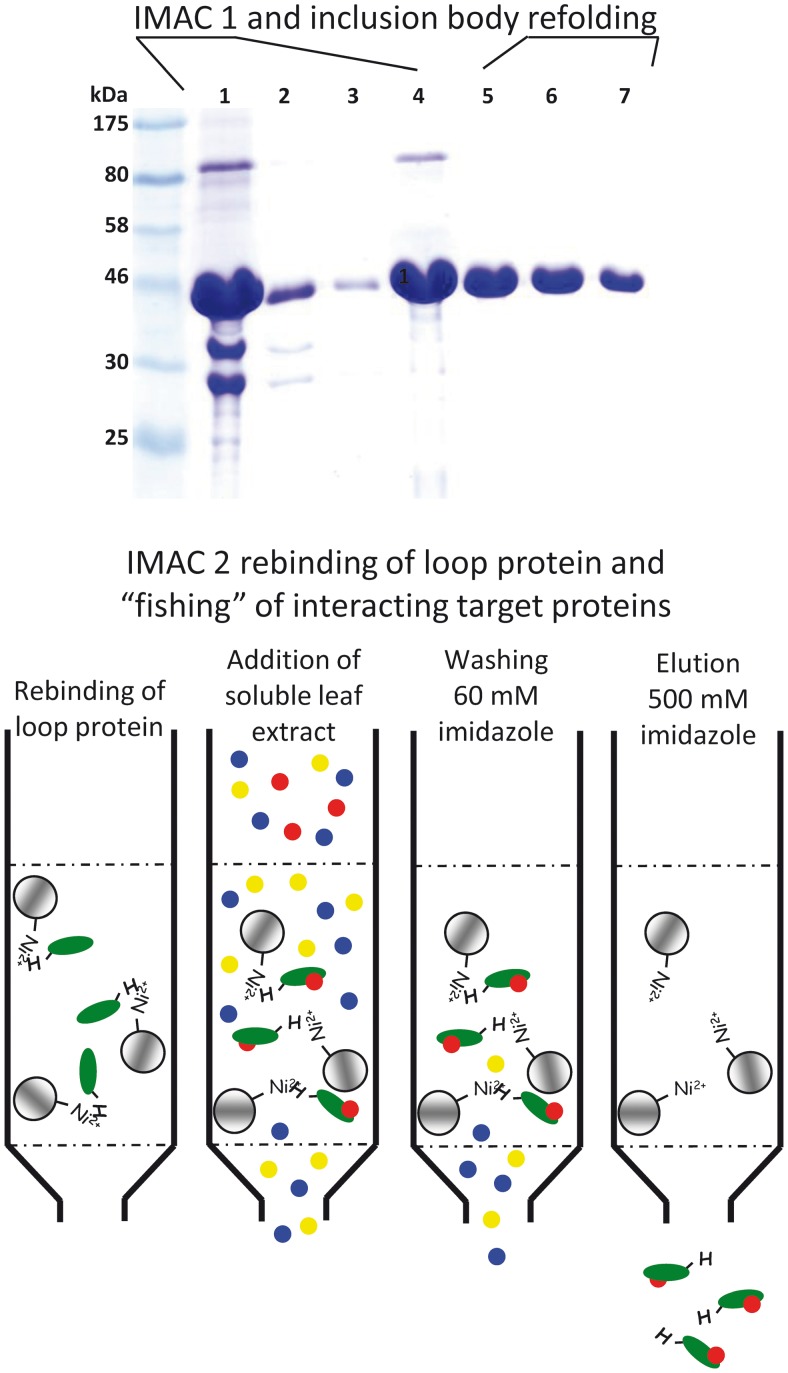
**Schematic drawing to illustrate the method applied for identification of TMT1 loop interacting *Arabidopsis* soluble proteins.** Upper part: IMAC 1. SDS-PAGE of protein samples of different TMT1 loop purification and refolding steps. (1) denatured (6 M urea) inclusion body fraction from *Escherichia coli* cells producing the TMT1 loop, (2) flowpath of the IMAC, (3) washing step, (4) eluted protein (still denatured), (5–7) samples from the stepwise removal of the denaturing agent by gel chromatography (Sephadex G25). Lower part: IMAC 2. Re-natured loop protein was coupled to Ni-Sepharose beads and a soluble *Arabidopsis* protein extract was added to the column. Subsequent to washing, loop and loop-interacting proteins were eluted by addition of 500 mM imidazole. Proteins in the eluate fraction were identified by mass spectrometry.

To identify protein–protein interactions different genetic strategies (e.g., yeast two-hybrid system for soluble proteins or the split ubiquitin system for membrane proteins) can be applied. However, it is sometimes more advisable to conduct a more goal-oriented approach. To identify proteins that might interact with the large hydrophilic TMT loop Wingenter et al. (2011) used the recombinant loop as bait (attached to beads) to fish for interacting proteins in a soluble *Arabidopsis* leaf extract (A schematic overview of the applied method is given in **Figure 1**). Attached proteins were analyzed by mass spectrometry (Wingenter et al., 2011). In total more than 90 proteins were identified, including the protein kinase VIK1, a member of the C1 group of MAPKKK (MAPK Group, 2002). Subsequent studies revealed that VIK1 physically interacts with the TMT1 protein *in vivo* and that it mediates TMT1 loop phosphorylation at least *in vitro*. Transport measurements with isolated *Arabidopsis* vacuoles either in presence or absence of recombinant VIK1 helped to clarify the impact of phosphorylation on TMT activity. Vacuolar glucose uptake was significantly stimulated by addition of ATP as phosphate donor and heterologously (*Escherichia coli*) expressed, purified VIK1. Furthermore, VIK1 *t*-DNA insertion lines phenocopy TMT loss-of-function mutants in important aspects: seedlings exhibit reduced growth and fresh weight in liquid media with high glucose concentrations (Wingenter et al., 2011). The observations that (i) recombinant VIK1 binds and phosphorylates the recombinant TMT-loop, (ii) presence of VIK1 stimulates vacuolar glucose uptake and (iii) TMT and VIK1 loss of function mutants are quite similar suggest that VIK1 mediated phosphorylation of the TMT-loop is a regulatory principle in vacuolar sugar import. However, the exact phosphorylation state of TMT proteins in VIK1 mutant plants has to be clarified by future attempts.

*Arabidopsis* mutants lacking TMTs show lower glucose and fructose uptake into the vacuole and significantly reduced (vacuolar) glucose and fructose contents compared to wild type plants. The differences in sugar transport and contents of wild type and mutant plants become more pronounced when plants are exposed to cold temperatures, an abiotic stress situation inducing cellular sugar accumulation and vacuolar sequestration (Wanner and Junttila, 1999; Wormit et al., 2006; Schulze et al., 2012). Accordingly, TMT-type transporters can be considered as the main glucose and fructose import systems of the tonoplast and therefore, it seems well justified to assume that TMT activity becomes increased upon cold temperatures. Interestingly, a proteomic study revealed a cold-induced increase in phosphorylation of a specific amino acid residue in the TMT1/2 loop (Schulze et al., 2012). However, until now it is not clarified whether VIK1 or other kinases mediate the cold-induced phosphorylation that causes the increased sugar uptake. Additional studies are required to identify the exact amino acid positions within the TMT loop that are targets of VIK1. Moreover, also the detailed impact of this post-translational modification on the biochemical properties of TMT proteins has to be analyzed.

**FIGURE 2:**
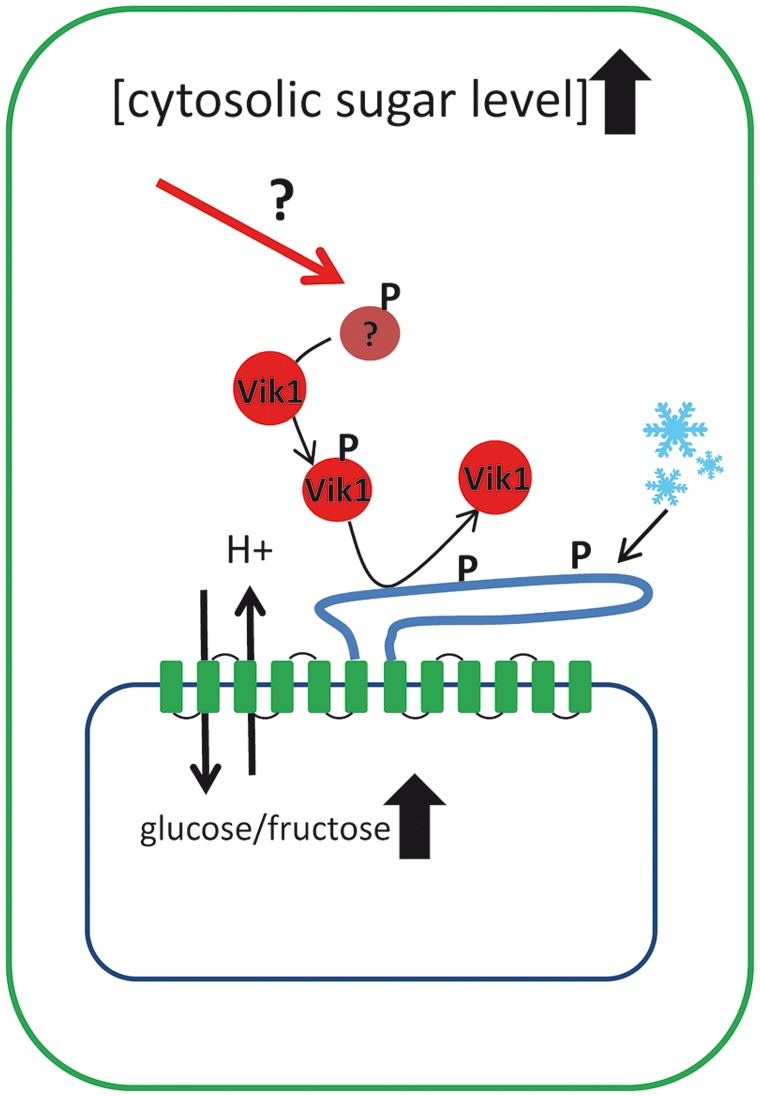
**Schematic drawing illustrating the current knowledge on how tonoplast monosaccharide transporters (TMTs) are regulated at the post-translational level.** TMT proteins represent the main monosaccharide transporters in the vacuole and possess an extraordinarily large loop between transmembrane domain 6 and 7. It has been shown that at least the loop region of TMT1/2 becomes phosphorylated upon cold acclimation. TMT phosphorylation is mediated by the MAPKKK VIK1 and its interaction with the TMT1 protein has been demonstrated under *in vivo* conditions. Presence of VIK1 stimulates glucose uptake into isolated vacuoles and VIK1 is capable of TMT phosphorylation under *in vitro* conditions. Apparently, upstream signals that regulate the activity of the VIK1, like cytosolic sugar concentrations, have to be identified in further studies.

In summary, these findings indicate that kinase mediated phosphorylation of TMT proteins is an important principle in adaptation of vacuolar sugar transport and in cellular sugar homeostasis (the current knowledge about TMT regulation is summarized in **Figure 2**). Of course these findings are only a first step in understanding the regulation of TMT-type proteins and continuing efforts are required to explain how TMTs are incorporated in the complex network of sugar sensing and sugar partitioning of plant cells. The TMT interacting kinase VIK1 belongs to the RAF related group C of MAPKKKs and within this group VIK1 is a member of the subgroup C1. All six members of the C1 subgroup (VIK1 and VIK1-like 1–5) exhibit an ankyrin repeat motif that is missing in all other MAPKKKs. Generally, ankyrin repeats are known to be involved in protein–protein interactions (MAPK Group, 2002). In future, several questions have to be answered: do (i) other members of C1 subgroup also interact with TMT proteins, (ii) do VIK1 and VIK1-like proteins interact with each other, and finally, (iii) how are VIK1 and maybe VIK1-like kinases embedded in the context of plant sugar sensing?

## STRESS INDUCED PHOSPHORYLATION OF TONOPLAST PROTEINS

High environmental salt concentrations represent an important abiotic stress situation for plants. However, plants have established different strategies to compensate for unfavorable salt accumulation in the cytosol. E.g., plasma membrane and tonoplast located proton-driven sodium export systems allow removal of sodium either into the apoplast or into the vacuole. The molecular principle of sodium secretion to the apoplast is quite well understood, it is mediated by the so called salt overly sensitive (SOS) pathway and comprises at least three proteins: the Na^+^/H^+^ antiporter (SOS1), the protein kinase (SOS2) that regulates SOS1 activity, and the calcium binding protein (SOS3) that on the other hand regulates SOS2 (Qiu et al., 2002). Apart from apoplastic salt secretion at least parts of the SOS pathway seem to be additionally involved in vacuolar salt sequestration. Sodium uptake studies with isolated tonoplast vesicles from SOS2 loss-of-function mutants led to the suggestion that presence of SOS2 is required to stimulate vacuolar sodium import activity via the tonoplast Na^+^/H^+^ antiporter (*At*NHX1; Qiu et al., 2004). However, direct evidence that *At*NHX1 becomes phosphorylated by SOS2 is missing. Nevertheless, in proteomic studies of *Arabidopsis* and barley phosphorylated NHX1 peptides were discovered (Whiteman et al., 2008b; Endler et al., 2009).

Interestingly, it has also been reported that SOS2 interacts with the vacuolar ATPase (V-ATPase; Batelli et al., 2007). This interaction was discovered by N-terminal fusion of an improved tandem affinity tag (NTAP) to SOS2, affinity purification and subsequent LC-MS/MS of the purified protein complexes. The corresponding data led to the suggestion that SOS2 mainly interacts with the peripheral V-ATPase subunits A, B, C, E, and G. Application of high salt concentrations significantly increased this interaction. *In vivo* interaction of SOS2 and V-ATPase subunits B1 and B2 were confirmed by yeast two-hybrid analyses (Batelli et al., 2007). However, the precise biochemical mechanism of how SOS2 triggers the activity of the V-ATPase remains unclear and the authors failed to demonstrate a putative SOS2 mediated phosphorylation of subunit B. Nevertheless, increased proton pumping activity during exposure to salt stress is of physiological advance because it drives and supports sodium uptake via the Na^+^/H^+^ antiporter (*At*NHX1) into the vacuole. The data underline the central role of SOS2 as a hub to activate transport proteins that remove Na^2+^ from the cytosol (either to the apoplast or the vacuole) and to activate proteins that provide energy for these transport activities.

Apart from a role in sodium sequestration it was also shown that the kinase SOS2 interacts with (and thereby activates) the vacuolar Ca^2+^/H^+^ antiporter CAX1 (Cheng et al., 2004). This interaction was demonstrated by the use of a Ca^2+^ hypersensitive yeast mutant that is unable to grow on high CaCl_2_ concentrations. Cheng et al. (2004) were able to complement this growth deficient phenotype by the co-expression of the *Arabidopsis* CAX1 and SOS2 in the corresponding yeast strain. Expression of CAX1 without SOS2 did not complement the phenotype. The protein–protein interaction of CAX1 and SOS2 was additionally demonstrated by yeast two-hybrid assays. The fact that SOS2 regulates sodium and also calcium uptake into the vacuole implies that cellular sodium and calcium homeostasis are interconnected systems.

Ca^2+^ is one central element in signal transduction and most abiotic, biotic but also hormonal stimuli cause changes in free cytosolic concentrations of this ion (Kudla et al., 2010). Exposure of *Arabidopsis* plants to high salt concentrations induces transient increase in free cytosolic Ca^2+^. Free cytosolic Ca^2+^ is sensed by at least three different types of proteins: (1) calmodulin, (2) calcineurin, and (3) calcium dependent protein kinases (Boudsocq and Sheen, 2013). The latter group of kinases is able to immediately convert the Ca^2+^ signal into phosphorylation of target proteins resulting in a modified activity of the proteins or enable binding of regulatory 14-3-3 proteins (Boudsocq and Sheen, 2013).

The large central vacuole represents a major reservoir for free Ca^2+^. In accordance with its function in plant defense against various stress situations the vacuole can also receive and react to cellular Ca^2+^ signals (Peiter, 2011). Tonoplast proteins were shown to directly interact with Ca^2+^-dependent protein kinases. One example of a Ca^2+^-dependent regulation of vacuolar solute passage is the selective tonoplast K^+^ channel TPK1 that becomes phosphorylated at a specific serine residue within the N-terminus by the Ca^2+^-dependent kinase CPK3. The corresponding modification supports interaction of TPK1 with GRF6, a 14-3-3 protein and this leads to channel activation (Latz et al., 2013). This regulation of TPK1 allows maintaining cytosolic potassium homeostasis, particularly in response to salt stress.

Identification of diverse phosphorylated tonoplast proteins in various plants and of several protein kinases that interact with tonoplast proteins and alter their activity clearly demonstrates that this type of post-translational modification is a regulatory principle concerning also tonoplast proteins. In the near future, the continually increasing sensitivity of mass spectrometry will help to detect a broad set of protein modifications and might allow comparative analyses of these protein modifications in plants that have been challenged with different stress situations.

## V-ATPases AND REGULATORY PROTEIN–PROTEIN INTERACTIONS

Transport of solutes across the tonoplast and particularly solute sequestration and concentration in the vacuole are energy consuming processes. Two types of proton pumps, the V-ATPase and the vacuolar proton pumping pyrophosphatase (V-PPase), use chemical energy of ATP or pyrophosphate to pump protons into the vacuolar lumen. Proton pumping activity causes considerable acidification of the vacuolar lumen and leads to establishment of a significant electrochemical gradient (of about 30 mV; Martinoia et al., 2007). According to the current knowledge plant vacuoles harbor both, V-ATPases and V-PPases, in parallel. However, in *Arabidopsis* V-ATPase activity is dominating during the phase of vegetative and reproductive growth whereas V-PPase activity seems to be required rather during gametophyte and embryo development (Krebs et al., 2010). Additionally, changes in abundance of V-ATPase and V-PPase have been studied in detail, e.g., in pear fruits (Shiratake et al., 1997). In young developing fruits the major proton pumping activity is provided by the V-PPase. During fruit development the activity of the V-ATPase increases constantly and represents the dominant proton pump in mature fruits. The importance of V-ATPase becomes supported by the fact that V-PPase activity cannot replenish proton pumping activity in plants lacking tonoplast located V-ATPase (Krebs et al., 2010). Corresponding *Arabidopsis* plants exhibit an immensely reduced acidification of the vacuolar lumen and consequently are also severely affected in nutrient storage.

For several years it was commonly assumed that – similar to V-ATPase – the V-PPase is mainly required for generation of a proton gradient across the tonoplast. Interestingly, when compared to V-ATPase the contribution of V-PPase to vacuolar acidification seems to be of minor relevance. Moreover, recent investigations suggest that main function of the V-PPase is hydrolysis and hence removal of cytosolic pyrophosphate and the associated proton pumping activity allows dissipation of the liberated energy. The corresponding pyrophosphatase activity is of substantial physiological importance during postembryonic heterotrophic growth (Ferjani et al., 2011).

The plant V-ATPase complex consists of several subunits. Generally, the complete complex can be divided into a peripheral entity (V1, with its respective subunits) that faces the cytosol and catalyzes ATP hydrolysis and a membrane spanning entity (V0, with its respective subunits) that performs proton transport. Each multimeric entity is composed by a subset of proteins that can vary in its composition. In *Arabidopsis* 14 genes encode different proteins that can build V1 and 14 genes encode proteins that can form the V0 entity. To harmonize the nomenclature of V-ATPase subunits in Sze et al. (2002) proposed to rename the genes encoding the subunits VHA-x, here x indicates the subunits: in lowercases for V0 subunits (from a to e) and in capitals for V1 subunits (from A to H). Diverse proteomic studies of the vacuole verified that V-ATPase is among the most abundant proteins in the tonoplast (Carter et al., 2004; Shimaoka et al., 2004; Jaquinod et al., 2007; Schulze et al., 2012). However, V-PPase and tonoplast intrinsic proteins (TIPs) also immensely contribute to the vacuolar protein fraction (Maeshima, 2001). Localization and function of V-ATPase are not restricted to the tonoplast (Schumacher and Krebs, 2010). The holoenzyme is detectable in most membranes accept from those surrounding plastids or mitochondria. Most likely assembly of the V1 and V0 subcomplexes takes place in the ER. From here the holoenzyme is targeted to its final destination, e.g., the trans-Golgi network/early endosomes (TGN/EE), the vacuole or the plasma membrane. Interestingly, V-ATPases of the TGN/EE contain the V0 subunit VHA-a1 whereas tonoplast complexes possess VHA-a2 or VHA-a3 (Schumacher and Krebs, 2010). Therefore, it can be assumed that the VHA-a component of V0 harbors information that determines whether the complete complex is targeted to the TGN/EE or to the tonoplast.

Increased demand of vacuolar metabolite storage requires increased activity of the tonoplast located V-ATPase. For example, during initiation of grape ripening (a process named “*vérasion*”) berries soften and start to accumulate massive amounts of glucose and fructose in the vacuole. Activity measurements and protein quantification revealed that both vacuolar proton pumps, V-PPase and V-ATPase, showed increased activity (Terrier et al., 2001). The corresponding increase in the proton gradient stimulates sugar uptake by proton antiporters.

Moreover, transfer of *Arabidopsis* plants to moderated cold temperatures induces the process of cold acclimation. The resulting increase in freezing tolerance is induced or at least accompanied by sequestration of compatible solutes (including sugars) in the vacuole. Cold acclimation was shown to increase abundance and hence activity of the V-ATPase resulting in higher vacuolar glucose and fructose uptake capacity (Schulze et al., 2012).

Apart from sugar accumulation and corresponding cold acclimation the vacuole might also be involved in salt stress management because (i) cytosolic sodium can be imported via the tonoplast Na^+^/H^+^ antiporter (*At*NHX1; Qiu et al., 2004), (ii) transcript and protein levels of the V-ATPase increase during salt stress, and (iii) V-ATPase activity is up-regulated by SOS2 in response to high environmental salinity (Batelli et al., 2007). These observations become complicated due to the fact that in *Arabidopsis* mutants lacking functional tonoplast V-ATPase (vha-a2 vha-a3 double mutant), sensitivity to salt stress remains rather unaffected whereas nutrient storage is markedly impaired. Moreover, reduced transcript levels of vha-a1 resulted in a SOS-like phenotype. These results suggest that the TGN/EE is important for sodium removal from the cytosol and that the energy of the corresponding transport process is provided by the TGN/EE located V-ATPase (Krebs et al., 2010).

Because of its role in balancing cytosolic levels of major metabolites and ions, and because of its function in different organelles the activity and subcellular distribution of the V-ATPase has to be tightly regulated. This can happen on transcriptional, translational, and post-translational level. There is rising evidence that this important proton pump is not only regulated by gene expression, also post-translational modification seems to be a relevant mechanism. However, up to now our knowledge on this type of V-ATPase modification is still incomplete.

In phosphoproteome studies of rice and barley phosphorylated peptides matching with subunits of the V-ATPase have been discovered (Whiteman et al., 2008a; Endler et al., 2009). Furthermore, interaction of V-ATPase subunits with at least three protein kinases has been experimentally demonstrated. Apart from SOS2 that apparently interacts with the peripheral subunits A, B, C, E, and G, a member of the kinase family WNK (*At*WNK8; with no K (lysine)) was shown to bind and phosphorylate subunit C at multiple sites (Hong-Hermesdorf et al., 2006). The relevance of this phosphorylation on the biochemical properties of the V-ATPase remains to be clarified. However, it is tempting to speculate that the modification might be involved in re-/disassembly of the V1 and V0 complexes of the V-ATPase, a well characterized regulation that adapts vacuolar energy supply and cellular pH homeostasis in yeast, insects and mammals (Qi et al., 2007). In aleuron cells of developing barley seedlings, a Ca^2+^-dependent protein kinase (CDPK) has been identified that activates V-ATPase and thereby stimulates vacuolar acidification. CDPK was shown to be involved in vacuolation in the endosperm but does not influence cytoplasmic Ca^2+^ increase or gene expression that is typically induced by gibberellic acid during endosperm reserve mobilization. Accordingly, CDPK is supposed to fulfill a specific function in tonoplast transport regulation in aleuron cells (McCubbin et al., 2004). The kinase possesses the capacity to phosphorylate tonoplast proteins. However, the molecular nature of the modified protein(s) is still unknown. It is imaginable that CDPK-mediated increase in V-ATPase activity is caused by phosphorylation of specific subunits.

Higher V-ATPase activity consequently leads to higher ATP consumption. Therefore, the question arises whether a regulatory connection between the ATP requiring process of proton pumping and ATP generating pathways exists. Quantitative proteome studies of tonoplast proteins purified from salt-stressed *Mesembryanthemum crystallinum* plants identified a possible interaction of two glycolytic enzymes, aldolase, and enolase, with the subunit VHA-B (Barkla et al., 2009). Moreover, gene expression and enzymatic activity of the corresponding aldolase and enolase were shown to be up-regulated after salt treatment. Furthermore, the authors were able to demonstrate that purified recombinant aldolase stimulates activity of V-ATPase by increasing its affinity to ATP. To underline the proposed interaction of V-ATPase and the glycolytic enzymes *Arabidopsis* mutant plants with reduced cytosolic enolase activity were analyzed subsequent to salt stress treatment. These mutant plants showed a reduced salt tolerance, a reduced aldolase depended stimulation of hydrolytic V-ATPase activity and reduced levels of tonoplast associated enolase. Interestingly, detailed investigation of tonoplast proteome data provided further indications for association and maybe interaction of further glycolytic enzymes with tonoplast proteins (Barkla et al., 2009). However, in the original proteome studies these glycolytic proteins were mostly classified as contaminations that occur during organelle/membrane preparation.

14-3-3 proteins are important elements in plant blue light perception and are known to interact with F-ATPases (Bunney et al., 2001). In Klychnikov et al. (2007) investigated a possible interaction of 14-3-3 proteins with the V-ATPase. They were able to demonstrate that short treatment of etiolated barley coleoptiles with blue light results in increased activity of a kinase that is capable of phosphorylation of the V-ATPase subunit A at least *in vitro*. The corresponding subunit subsequently interacts with 14-3-3 proteins resulting in a higher proton pumping activity (Klychnikov et al., 2007).

Hexokinase1 (HXK1) fulfills diverse signaling functions particularly in regulation of gene expression and hence might be regarded as the most important glucose sensor of plants. In *Arabidopsis* HXK1 is mainly present in the cytosol. However, a minor degree was also detected in the nucleus. In the nucleus this minor portion of HXK1 interacts with a 19S regulatory particle of the proteasome subunit (RPTB5) and with the B1 subunit of the V-ATPase (VHA-B1). The corresponding proteins (HXK1, VHA-B1, and RPTB5) form a complex that exclusively occurs in the nucleus. Moreover, no interaction with other V-ATPase subunits, not even with the relative homologous forms of VHA-B1 (VHA-B2 or VHA-B3) could be identified. This observation suggests that nuclear located interaction of HXK1, VHA-B1, and RPTB5 is highly specific (Cho et al., 2006). Therefore, subunit VHA-B1 is assumed to fulfill an additional, totally unexpected function besides its role as a component of the proton pump. VHA-B1 and RPTB5 loss of function mutants exhibit a glucose insensitive (*gin*) phenotype comparable to that of HXK1 mutants (originally named *gin2* mutant). Accordingly, the nuclear complex of the three proteins might be physiologically relevant for glucose sensing and related signaling pathways. Furthermore, the function of VHA-B1 in glucose signaling seems to be independent of its role in V-ATPase function because other VHA mutants (vha-A9 and vha-E1) are lethal (Dettmer et al., 2005; Strompen et al., 2005) or absence of the subunits resulted in a highly glucose sensitive phenotype (det3/vha-C; Cho et al., 2006).

Taken together these analyses provide evidence that the V-ATPase is regulated by diverse mechanisms and that at least one subunit of the complex has an additional, independent regulatory function.

*In vitro* studies revealed that V-ATPases of plants as well as those from mammals and fungi can be inactivated by oxidizing agents (e.g., H_2_O_2_) and that the activity can be restored by reducing agents. The subunit A contains three cysteine residues (conserved in all eukaryotes) that have been proposed to be the target for this redox modulation. Indirect evidence suggests that redox modulation of the V-ATPase also occurs *in vivo* because yeast mutants with impaired glutathione biosynthesis shows reduced proton pumping activity. In Seidel et al. (2012) analyzed the proposed redox modulation of the V-ATPase from *Arabidopsis* in detail. Mutation of the three conserved cysteine residues of VHA-A to serine and complementation of a *VHA-A* null mutant with the modified *VHA-A* genes revealed that oxidative inhibition does not represent a primary regulatory mechanism in plant V-ATPases. Mutations of cysteine residues 256 and 535 to serine in VHA-A resulted in a fully complemented vha-a phenotype comparable to that of wild type plants. Solely, mutation of cysteine 279 to serine did not fully compensate the impaired growth phenotype. Even under conditions of a fully oxidized glutathione pool in root cells, corresponding vacuolar pH was totally unaffected in all complementation lines (Seidel et al., 2012). These findings differ from those obtained with the glutathione biosynthesis deficient yeast strain and support the author’s conclusion that oxidative inhibition is not a principle common among V-ATPases from different organisms (Seidel et al., 2012).

In sum, the given examples suggest that the complexity of the diverse functions of V-ATPases in different compartments and in a broad variety of metabolic and physiological processes requires complex regulation of expression, synthesis, assembly, localization, and activity of the respective subunits. Changes in expression of genes encoding the different subunits correlate with changes in environmental parameters like salt, osmotic, chilling, heat, and drought stress. Moreover, expression of certain subunits varies in a tissue-specific manner (e.g., vha-E vha-G) whereas expression of other subunit genes seems to be rather tissue-independent (vha-a). There is also increasing evidence for V-ATPase regulation by post-translational modification (phosphorylation), by interaction with regulatory proteins (SOS2, WNK8, CDPK, 14-3-3 proteins) or enzymes generally known from primary metabolism (enolase, aldolase). Other regulatory principles that have been described for V-ATPases from yeast, insects and mammals have not been observed yet or are of minor importance in plants (redox modulation). Different targeting of subunits can also be seen as a mechanism of regulation (vha-a1 versus vha-a2 and3). The detailed complexity of subunit targeting and assembly in different cellular membranes remains to be addressed in future studies. Finally, subunit B1 in complex with HXK1 and RPTB5 exhibits a regulatory function in nuclear gene expression that is apparently independent of the proton pumping activity of the holoenzyme.

## CONCLUSION AND OUTLOOK

Tonoplast proteins and accordingly vacuolar transport processes are regulated by diverse mechanisms and by this become adapted to the specifically required function of the vacuole. Transcriptional regulation, increase or decrease in protein abundance and also post-translational modifications were discovered for diverse tonoplast proteins.

Although necessity for tight control and regulation of tonoplast transport is quite apparent, comprehensive and precise investigations are mandatory to decipher the complete regulatory network and to clarify the specific impact of the regulatory proteins. Biochemical relevance and physiological consequences of tonoplast protein modifications and protein interactions have to be analyzed in more detail. Sophisticated comparative proteome analyses from “control” plants and from plants challenged with different stressors or exposed to environmental changes will uncover associated alterations in post-translational modification (phosphorylation, etc.). Subsequently, the impact of the identified modification on the protein activity has to be clarified (e.g., by *in vitro* activity measurements, mutant proteins, mutant plants lacking the respective kinase, etc.). Although progress has been made concerning phosphorylation of tonoplast proteins specific important details are missing. For example, interaction of the kinase VIK1 with the TMT protein, VIK1 mediated phosphorylation of the TMT loop and stimulation of TMT activity due to presence of VIK1 have been demonstrated, however, it is still unclear which specific TMT amino acid residue(s) represent the actual targets of VIK1 and which alterations of the biochemical properties are induced by specific modifications the TMTs. Interestingly, there is also evidence that TMT proteins are capable of sucrose transport, identified by patch clamping of isolated *Arabidopsis* vacuoles (Schulz et al., 2011), and it is tempting to speculate that the affinity of TMT proteins for mono- or disaccharides becomes modified by kinase activity. However, determination of phosphorylation and identification of a corresponding protein kinase always leads to several new questions because a kinase mediated signal usually is only one step in a signaling cascade. For example VIK1 apparently represents the final kinase directly interacting with the target transport protein. The next challenge will be the identification of upstream components in the signaling network.

## Conflict of Interest Statement

The authors declare that the research was conducted in the absence of any commercial or financial relationships that could be construed as a potential conflict of interest.
